# *Candida* fracture-related infection: a systematic review

**DOI:** 10.5194/jbji-6-321-2021

**Published:** 2021-08-23

**Authors:** Daniele De Meo, Gianluca Cera, Giancarlo Ceccarelli, Valerio Castagna, Raissa Aronica, Edoardo M. Pieracci, Pietro Persiani, Ciro Villani

**Affiliations:** 1 Department of Anatomical, Histological, Forensic Medicine and Orthopaedic Science, Sapienza University of Rome, Piazzale A. Moro 5, 00155, Rome, Italy; 2 M.I.T.O. Study Group (Infectious Diseases in Traumatology and Orthopedics Surgery), Policlinico Umberto I University Hospital, Viale del Policlinico, 155,00161 Rome, Italy; 3 Department of Public Health and Infectious Diseases, Sapienza University of Rome, Piazzale A. Moro 5, 00155, Rome, Italy

## Abstract

**Background**: The aim of this study is to summarize and improve knowledge regarding a *Candida* fracture-related infection (CFRI) through a systematic review on the topic, accompanied by a case report. **Methods**: A systematic review and meta-analysis based on PRISMA statement were conducted on the CFRI topic. The following combined search terms were used to explore PubMed, Cochrane, and the Embase database: “fungal infection”, “candida”, “fracture related infection”, “bone infection”, “orthopedic infection”, “internal fixation”, “post-traumatic infection”, and “osteomyelitis”. **Results**: Out of 1514 records, only 5 case reports matched the selection criteria and were included. Moreover, a new case of CFRI, not previously described, was reported in this paper and reviewed. The main risk factors for CFRI were open wounds (three cases) and immunodeficiency (three cases). Initial improvement of clinical and laboratory signs of infection was noted in all cases. In the available short-term follow-up (mean 12.1 months; range 3–42), the reoperation rate was 33.3 %. Using a strategy based on extensive debridement/resection methods and prolonged systemic antifungal therapy (mean 8.8 weeks; range 6–18), four of six cases (66.6 %) were cured. Bone union occurred in three out of six cases. **Conclusion**: There is very low-quality evidence available regarding CFRI. *Candida* infections in surgically treated fractures are rare but difficult-to-treat events, with a slow onset, unspecific symptoms or signs, and a significant relapse risk; therefore, they still represent a current diagnostic challenge. The existing fracture-related infection treatment algorithm combined with long-term systemic antifungal therapy has an anecdotal value and needs more extensive studies to be validated.

## 1 Introduction

*Candida* infections are extremely rare events in orthopaedic patients. Hematogenous osteomyelitis is the most frequent type of these infections, often located in the vertebrae (Gamaletsou et al., 2012). The current literature has been raising interest of this type of bone and joint infection, also in the prosthetic joint infection field (Cobo et al., 2017; Riaz et al., 2020). However, there are poor indications for a *Candida* fracture-related infection (CFRI), which are reported only as a series of cases. The purpose of this study was to conduct a systematic review of the published literature on previously reported cases of CFRI, identifying risk factors, diagnosis, and treatment strategies, in order to summarize and improve data regarding this uncommon infection. Furthermore, we reported an unusual clinical case of CFRI that was treated in our institution.

## 2 Materials and methods

The definition of fracture-related infection (FRI) was compliant with criteria recently reported by McNally et al. (2020); the *Candida* etiology of FRI was based on the isolate of the microorganism in adequate and representative bone specimens related to the fracture (McNally et al., 2020).

**Figure 1:**
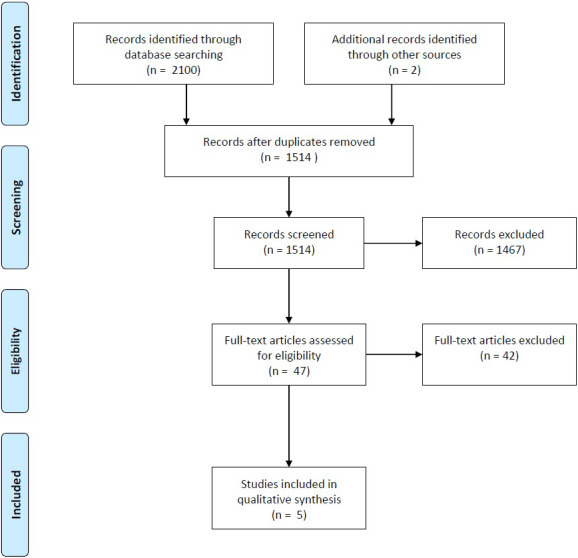
PRISMA flow diagram of the CFRI systematic review.

The authors followed the Preferred Reporting Items for Systematic Reviews and Meta-Analyses (PRISMA) statement when conducting and reporting this systematic review. In May 2021, the PubMed, Cochrane, and Embase databases were searched. The following combined search terms were used: “fungal infection”, “candida”, “fracture related infection”, “bone infection”, “orthopedic infection”, “internal fixation”, “post-traumatic infection”, and “osteomyelitis”. The inclusion criteria were the following: English language articles, fracture-related infections, available data on documentation of *Candida* osteomyelitis with direct microbiologic or histopathologic evidence, anatomical location of infection, underlying condition, and therapeutic intervention. The exclusion criteria were prosthesis joint infection and bone infection not clearly related to fractures. Four authors (Gianluca Cera, Valerio Castagna, Raissa Aronica and Edoardo M. Pieracci) separately assessed all the identified publications for eligibility, after which one of the authors (Daniele De Meo) reviewed the results and approved the selected studies based on the aforementioned criteria (Fig. 1). This study also includes one case report of a patient who developed CFRI. The patient's informed consent was obtained.

## 3 Results

### 3.1 Literature review

Of the initial 1514 records, only 5 case reports regarding CFRI were found in the literature (Lopez et al., 2014; Goff et al., 2014; Machi et al., 1994; Oliverson et al., 2002; Yingling et al., 2017). The data related to said cases can be found in Table 1. Two other articles containing CFRI were present in the literature but were excluded due to the impossibility of extrapolating the specific data of the cases searched (Miller et al., 2015; Tan et al., 2018). Six patients, including our case, are reported (four male and two female) with a mean age of 47.6 years. The main risk factors for CFRI were open fractures (three cases) and immunodeficiency (three cases). The infection site was the lower limb in five cases (two in the femur, two in the tibia, one in the fibula) and a phalanx of the hand in one case.

In three cases the fracture was open. In all cases, the fracture was surgically treated with the implantation of an internal fracture fixation device. The time between trauma and diagnosis was variable, ranging from 2 weeks to 21 years. The most frequent clinical sign was communication between the skin and the bone plane, present in 83.3 % (five cases). The species of *Candida* isolated were *C. albicans* in two cases, *C. parapsilosis* in three cases, and *C. glabrata* in one case. In three of the cases, other microorganisms were isolated along with the *Candida*.

All patients underwent targeted antifungal therapy for a mean of 8.8 weeks (range 6–18) and surgical treatment for the infection: in all cases except for one, the hardware implanted prior to the onset of the infection was removed. Initial improvement of clinical and laboratory signs of infection was noted in all cases. The average follow-up available was 12.1 months (range 3–42). In this observation period, the reoperation rate was 33.3 %.

Limiting to the available follow-up, four of six cases (66.6 %) were out of relapse, using a strategy based on extensive debridement/resection methods and prolonged systemic antifungal therapy.

**Table 1:** Clinical features of reported cases of CFRI in the literature. MDR: multi-drug resistant; CHF: congestive heart failure; CKF: chronic kidney failure. NA – not available.

Reference	Patient age/sex	Medical background	Trauma-related conditions	Site	Time between trauma and diagnoses (weeks)	Clinical signs	CRP	ESR	Pathogen	Other pathogens
Case report	52/F	Smoker, breast cancer,chemotherapy	Plate breakage	Tibia	117	Plate exposure	Neg	Neg	*C. albicans*	*S. aureus* MDR
Lopez et al.(2014)	51/M	None	Factory trauma open fracture	Phalanx (hand)	2	Purulent wound discharge	Neg	Neg	*C. parapsilosis*	None
Goff et al.(2014)	31/M	None	Lung contusions intensive care unit prolonged stay	Femur	49	Pain	12	11	*C. albicans*	Coagulase-negative staphylococcus
Machi et al.(1994)	70/M	Diabetes, gastric cancer	Open fracture	Femur	35	Pain, purulent discharge	4.7	117	*C. glabrata*	None
Oliverson etal. (2002)	34/M	smoker	Open fracture	Tibia	16	Sinus tract	NA	NA	*C. parapsilosis*	None
Yingling etal. (2017)	NA/F	HIV, HBV, HCV CHF, CKF cerebral vascular accidents dementia	None	Fibula	21 years	Plate exposure Septic shock	NA	NA	*C. parapsilosis*	*Proteus mirabilis*

**Table 2:** Treatment features of reported cases of CFRI in the literature. EF: external fixator; IM: intra-medullary; HWR: hardware removal; DBM: debridement; BMP: bone morphogenetic protein; OD: once daily; BID: twice a day; TID: three times a day; AMP-B: amphotericin-B deoxycholate; FCZ: fluconazole. NA – not available.

Reference	Surgical treatment	First stage	Second stage	Antibiotic therapy	Therapy duration (weeks)	Reoperation	Bone union	Infection outcome	Follow-up (months)
Case report	Yes	HWR + DBM + distraction osteogenesis with EF	EF removal + IM nailing + docking site DBM.	FCZ 400 mg OD + levofloxacin 750 mg	18	1	No	Relapse	15
Lopez et al.(2014)	Yes	HWR + DBM	–	Ceftriaxon 2 g OD + Rifampicin 300 mg BID until culture results. Caspofungin 50 mg OD, then FCZ 400 mg OD	6	0	Yes	Cured	3
Goff et al.(2014)	Yes	DBM + HWR +PMMA spacer (gentamicin + vancomycin + amphotericin B) +bridging EF	DBM + cement removal + morsellized autologous graft (RIA) + BMP-7 + blade plate	FCZ 50 mg OD + amoxicillin 1 g TID	6	0	Yes	Cured	42
Machi et al.(1994)	Yes	HWR + DBM + EF	–	AMP-B (1084 mg) + Flucytosine (100 mg/kg OD)	6	0	Yes	Cured	10
Oliverson etal. (2002)	Yes	DBM + cement beads (amphotericin B)	DBM + cement beads removal + bone graft	FCZ 400 mg OD for 8 weeks	8	0	No	Cured	3
Yingling etal. (2017)	Yes	HWR + DBM	DBM + negativepressure wound therapy dressing	FCZ, then caspofungin	NA	1	NA	Deceased during hospitalization	NA

### 3.2 Case report

A 52-year-old smoker Caucasian woman suffered closed fractures of the distal tibia and fibula due to a car–pedestrian collision in December 2014; initial treatment was an external fixation (EF) replaced with an open reduction with a locking plate fixation on the tibia, in March 2015. A surgical site infection due to methicillin-resistant *Staphylococcus aureus* (MRSA) was diagnosed 1 month after the surgery: for this reason, the patient was treated by plate and screws removal, targeted antibiotic therapy, and multiple surgical debridement with loss of soft tissue and subsequent coverage with a soleus flap. Finally in January 2016, the patient underwent bone graft and stabilization with plate and screws on the tibia. At the same time, she received a new diagnosis of malignant breast neoplasia and, after a mastectomy, chemotherapy was started.

**Figure 2:**
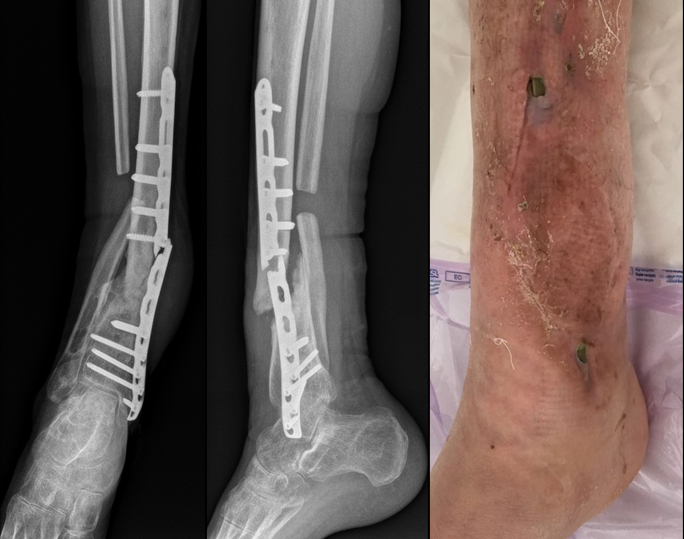
Radiographs and clinical pictures upon admission, in CFRI case report.

**Figure 3:**
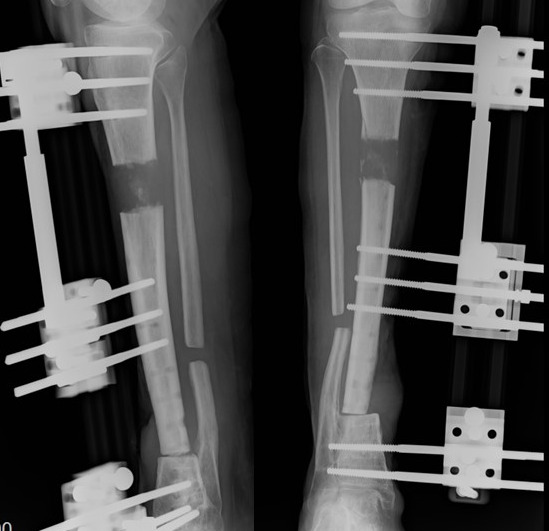
Follow-up X-ray during bone lengthening, after debridement, in CFRI case report.

**Figure 4:**
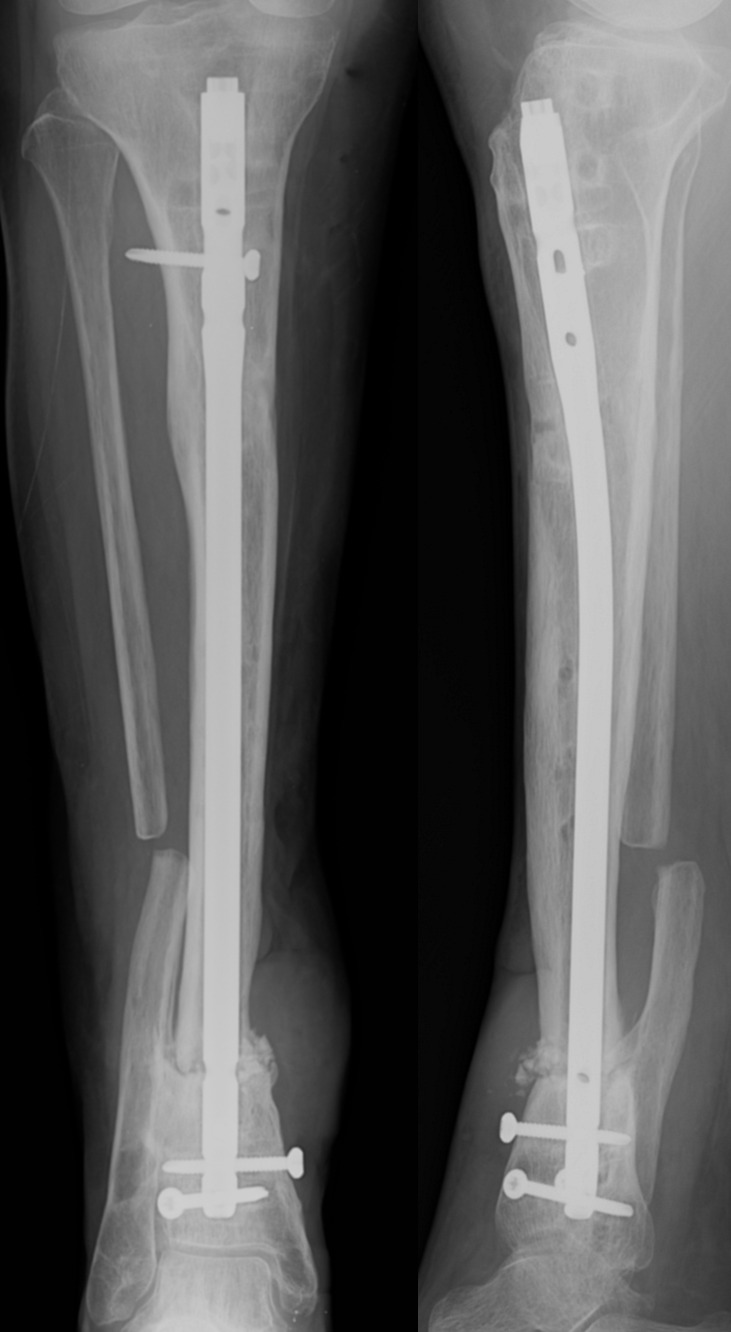
Follow-up X-ray after nail dynamization, in CFRI case report. Failed union at distal tibia with an osteolytic bone cyst in tibia, sclerotic lines around the distal screws.

In May 2017, the patients immunosuppressed due to chemotherapy, suffered by a spontaneous break and exposition of the plate (Fig. 2). Hardware removal and debridement procedures were performed, the nonunion site was completely resected, and the screw holes next to the nonunion site were debrided. A distraction osteogenesis was achieved by means of EF. A *Candida albicans* infection was isolated via plate sonication and confirmed by means of one bone sample isolation; a systemic dissemination of the *Candida* infection was excluded by blood cultures, eyes fundus evaluation, and echocardiogram. Targeted therapy with fluconazole 400 mg per day (OD) was administered for 18 weeks. In August 2017, the elongation was complete (initial gap 4 cm approx.) (Fig. 3). In May 2018, there was good bone regeneration in the proximal tibia elongation site though there was a docking site nonunion, with no soft tissue inflammation. C-reactive protein (CRP), erythrocyte sedimentation rate (ESR), and labelled leukocyte scintigraphy were negative. Full-weight load bearing was allowed with no pain. The EF was then removed followed by gentamicin-coated intramedullary tibial nailing (ETN PROtect™ – DePuy Synthes Companies, Zuchwil, Switzerland) and bone grafting in the nonunion site. Standard systemic antibiotic prophylaxis with cefazoline was administered. The microbiological and histological intraoperative samples of the nonunion site were negative for infection. The wounds healed uneventfully. In August 2018, an X-ray examination revealed persistent bone healing delay; thus a proximal nail dynamization was re-performed. Two weeks after the dynamization, the patient returned to the outpatient clinic due to pain and swelling in the docking site (Fig. 4). Once again, sinus tract was present and there were no signs of bone healing progression. The patient refused other surgeries or treatments and never returned.

## 4 Discussion

### 4.1 Epidemiology and clinical features

CFRIs are most frequently caused by *C. parapsilosis* and *C. albicans*. *C. albicans* is the most common species isolated; other species are *C. tropicalis*, *C. parapsilosis*, and *C. glabrata* in vertebral hematogenous osteomyelitis and other *Candida* osteomyelitis (Gamaletsou et al., 2012; Neofytos et al., 2014; Slenker et al., 2012). Half of the patients of this series had a polymicrobial FRI, a high percentage consistent with the results of previous data in the literature (McNally et al., 2020; Jorge et al., 2018). The hematogenous dissemination pattern seems to be the main mechanism of *Candida* bone and joint infections (67 %), followed by direct inoculation (25 %) and contiguous infection (9 %) (Gamaletsou et al., 2012). In CFRIs, direct inoculation seems to be the main pattern, considering the post-surgical nature of all infections discussed here.

According to the critical review preformed here, the main predisposing CFRI factors could be open fractures, immunosuppression, and prolonged broad-spectrum antibiotic therapy (Lopez et al., 2014; Goff et al., 2014; Machi et al., 1994; Oliverson et al., 2002; Yingling et al., 2017). The patient reported in this case report was a smoker with breast cancer, treated with surgery and systemic chemotherapy. All already known risk factors for *Candida* hematogenous osteomyelitis are burns, intravenous drug abuse, central venous access catheters, diabetes, < 1 year prior surgery at the infection site, prolonged broad-spectrum antibiotic therapy, intensive care unit, neutropenia, total parenteral nutrition, immunosuppression, liver disease, and human immunodeficiency virus (Mullins et al., 1993; Gathe et al., 1987; Lasday et al., 1994; Slenker et al., 2012).

The CFRI's clinical features in our systematic review were purulent discharge, sinus tract, local pain, a burning sensation, fever, and diminished deep tendon reflex. With regard to the blood inflammatory markers, in most of patients the white cell count and C-reactive protein did not increase at any stage of the infection. These data seem to overlap with the other types of *Candida* bone and joint infections, showing an insidious and heterogeneous onset. As a matter of fact, main *Candida* hematogenous osteomyelitis manifestations are local pain with confirmatory tenderness, erythema, edema, limitation of functions and movements, draining of pus, or sinus tracts. According to Gamaletsou's review (2012), fever is present only in a small number of patients, the onset is frequently insidious, starting with local symptoms and developing from subacute to chronic course, with a moderate or minimal response of the laboratory inflammation biomarkers.

The diagnosis of *Candida* osteomyelitis is based on clinical and laboratory data. Blood inflammation markers are useful for diagnostic purposes in early stages, but they are non-specific.

### 4.2 Laboratory diagnosis

With the introduction of the definition “fracture-related infection”, well-defined diagnostic criteria have been delineated based on confirmation criteria and suggestive criteria of FRI (Metsemakers et al., 2018). The diagnosis is then actually based on clinical criteria and intraoperative sampling for microbiological cultures and histopathological analysis, along with the sonication of the explanted hardware (Onsea et al., 2018).

In *Candida* bone and joint infections, a study to exclude candidemia and potential metastatic localization must be carried out with serial blood cultures, ophthalmic examination, and cardiac ultrasound imaging (Pappas el al., 2016; Vinikoor et al., 2013). The value of the inflammation indices can be fallacious and do not provide useful information to monitor the presence of a *Candida* infection and/or its relapse. On the other hand, (1,3)-β-D-Glucan blood test could provide useful information related to the persistence of the infection (Pappas el al., 2016; Vinikoor et al., 2013).

### 4.3 Surgical treatment

In this CFRI review, implant removal and debridement procedures were performed in all cases. EF was used in cases where there was residual instability of the bone segment after debridement. Antibiotic-loaded bone cement was used as a dead space management tool and local delivery of antibiotics in two cases. A second surgical stage was needed in four cases; bone healing occurs in only three out of six cases. In the two phalanx cases there was no need for further surgical interventions and no other implants were necessary. In the case of Oliverson et al. (2002), the patient underwent two surgical debridements with cement bead placement and a third operation for bead removal and bone grafting. In Machi's case, debridement with hardware removal and EF positioning led to a full resolution of the infection and nonunion (Machi et al., 1994). In Goff's report, a two-staged strategy with the induced membrane technique led to a fully functional recovery (Calori et al., 2011; Goff et al., 2014). In our case, the distraction osteogenesis technique was applied; it has the advantage of being a theoretic one-stage treatment, limiting the patient's surgical events and with no device left implanted. The main limit of this technique is a possible consolidation delay at the docking site (Zhang et al., 2016); if it occurs, as in this case, further surgery is required (Biz et al., 2014). According to the current management of chronic bone and joint infection and fracture-related infections, a staged approach with aggressive debridement, implant removal, and bone and soft tissue reconstruction is necessary (Depypere et al., 2019). The insidious and clinically indolent course of CFRI may be an additional factor, in our opinion, in the already known difficulty of bone healing of fracture-related infections (Foster et al., 2021).

Marra et al. (2001), along with two cases of the review (Oliverson et al., 2002; Goff et al., 2014), described a safe use of amphotericin B added to plain bone cement to act as a local antibiotic treatment. It has been used successfully by other authors to treat periprosthetic joint infections (Escola-Verge et al., 2018). Local antibiotic placement could be useful also in these patients if combined with Masquelet's technique. Local antifungal therapy strategies could be used to protect the new implant without the need to apply antibiotic cement, using antibiotic-coated gels and coatings, already proven effective in the prevention of infections (De Meo et al., 2020) and in the treatment of bacterial infections (De Meo et al., 2020).

To date, fracture-related infection treatment is based on initial surgical treatment, followed by targeted antibiotic therapy.

The failure of our case may be due to several factors: the patient underwent multiple surgeries prior to the diagnosis of the infection; failure to use targeted local antibiotic therapy against *Candida* during nail implantation; poor vascularization due to a deficient soft tissue envelope that was subjected to numerous surgical procedures, the duration of systemic antifungal therapy that could have been extended 6–12 months, considering a treatment time for osteomyelitis.

### 4.4 Antifungal treatment

A wide range of antifungal pharmacological strategies have been proposed: amphotericin-B deoxycholate was the most common antifungal agent used. The prescriptions of fluconazole 400 mg OD for 6–12 months or an echinocandin regimen in the first two weeks, followed by 6–12 months of fluconazole, were also suggested (Pappas et al., 2016).

Some authors recommend a shorter time of antifungal treatment after proper debridement in selected patients (Lopez et al., 2014; Cho et al., 2010). In a wider review regarding *Candida* osteomyelitis, Slenker et al. (2012) conclude that surgery did not appear to have a significant impact on therapy duration. More recently, experts pointed out that an inadequate length of therapy seems to be the most common cause of relapses (Henry et al., 2017). However, this statement is based on heterogeneous groups of *Candida* bone infection patients in terms of antifungal treatment, surgical strategies, infection sites, and transmission.

Regarding the adequate duration of antifungal therapy, currently available data are still anecdotal, and the level of evidence is very low being principally based on case reports or case series. However, it should be emphasized that *Candida* is a potentially slow-developing pathogen that is difficult to eradicate in the setting of fracture-related infections. The behaviour of *Candida* bone and joint infections needs a careful, long-term follow-up, to assess the possibility of a slow onset of relapses. The best antifungal treatment and the duration of antifungal treatment are still unclear, and the utility of chronic antifungal suppressive therapy is still controversial, although it has been used in other clinical settings (Ioannou et al., 2020).

### 4.5 Limits

This review is burdened by a number of major limitations. In particular data were obtained from case series, and for this reason the level of evidence available is very low. Moreover, the search strategy was based on an algorithm with a limited key word selection introducing a possible bias of sampling. Finally, a full comparison of clinical data reported and therapeutic strategy adopted is not possible, according to differences of variables used in the different available reports. The critical issues presented are the main ones, but nevertheless they do not encompass all methodological issues, due to a widely heterogeneous selection sample.

## 5 Conclusions

CFRIs are poorly reported in the literature when compared to *Candida* periprosthetic joint infections and vertebral hematogenous osteomyelitis. To the best of our knowledge, there are no reviews in the literature regarding CFRI.

Surgeons should be aware that persisting delayed healing or persisting non-unions harbour the risk of a fungal infection complication. However, there is no consensus about the necessity of surgical intervention or surgical strategies. If adopted, the surgical approach should be aggressive, combining debridement, hardware removal, and reconstruction techniques in the case of large bone defects. It must also aim at obtaining an etiological diagnosis, with adequate sampling for microbiological studies.

As for *Candida* osteomyelitis, the concomitant antifungal treatment should be possibly targeted and prolonged (suggested 3–12 months) to reduce the possibility of a slow onset of relapses. Accordingly, it is necessary to distinguish fracture-related infection even in *Candida* bone infection settings, due to specific features of the pathogenic process. Further large studies are needed to address the lack of specific information on the challenging topic of CFRI.

## Data Availability

The data used to support the findings of this study are included in the article (Sect. 3.1 and 3.2, Tables 1 and 2).
